# Contribution of Diffusion-Weighted Imaging and ADC Values to Papillary Breast Lesions

**DOI:** 10.3389/fonc.2022.911790

**Published:** 2022-06-30

**Authors:** Wenjie Lv, Dawen Zheng, Wenbin Guan, Ping Wu

**Affiliations:** ^1^ Department of Breast Surgery, Xinhua Hospital Affiliated to Shanghai Jiao Tong University School of Medicine, Shanghai, China; ^2^ Department of General Surgery, Xinhua Hospital Affiliated to Shanghai Jiao Tong University School of Medicine, Shanghai, China; ^3^ Department of Pathology, Xinhua Hospital Affiliated to Shanghai Jiao Tong University School of Medicine, Shanghai, China

**Keywords:** diffusion-weighted imaging, apparent diffusion coefficient values, papillary breast lesions, magnetic resonance imaging, mass enhancement, non-mass enhancement, receiver operating characteristic curve

## Abstract

This study aimed to evaluate the role of apparent diffusion coefficient (ADC) values obtained from diffusion-weighted imaging (DWI) in the differentiation of malignant from benign papillary breast lesions. The magnetic resonance imaging (MRI) data of 94 breast papillary lesions confirmed by pathology were retrospectively analyzed. The differences in ADC values of papillary lesions under different enhancements in MRI and different pathological types were investigated, and the ADC threshold was determined by the receiver operating characteristic curve for its potential diagnostic value. The mean ADC values in borderline and malignant lesions (1.01 ± 0.20 × 10^-3^ mm^2^/s) were significantly lower compared to benign lesions (1.21 ± 0.27 × 10^-3^ mm^2^/s) (*P* < 0.05). The optimal threshold of the ADC value could be 1.00 × 10^-3^ mm^2^/s. The ADC values were statistically significant in differentiating between benign and malignant papillary lesions whether in mass or non-mass enhancement (*P* < 0.05). However, there were no statistical differences in the ADC values among borderline or any other histological subtypes of malignant lesions (*P* > 0.05). Measuring ADC values from DWI can be used to identify benign and malignant breast papillary lesions. The diagnostic performance of the ADC value in identifying benign and malignant breast lesions is not affected by the way of lesion enhancement. However, it shows no use for differential diagnosis among malignant lesion subtypes for now. The ADC value of 1.00 × 10^-3^ mm^2^/s can be used as the most appropriate threshold for distinguishing between benign and malignant breast papillary lesions.

## Introduction

Papillary breast lesions indicate a heterogeneous group of diseases including benign intraductal papilloma (IDP), borderline intraductal papilloma with atypical hyperplasia [intraductal papilloma with atypical ductal hyperplasia (ADH)], and malignant papillary lesions. Intraductal papilloma with ductal carcinoma *in situ* (intraductal papilloma with DCIS), papillary ductal carcinoma *in situ* (papillary DCIS), encapsulated papillary carcinoma (EPC), solid papillary carcinoma (SPC), and invasive papillary carcinoma (IPC) fall into the third category (1). Papillary protrusions with a dendritic fibrovascular stroma represent the general histopathological feature of papillary breast lesions (2).

Magnetic resonance imaging (MRI) is widely applied in detecting papillary breast lesions as a prominently viable imaging modality. Due to the diversity of pathological subtypes, the variability among observational factors in MRI, such as morphology feature, enhancement mode, and time–signal intensity curve, and coupled with the absence of evidence from large samples or prospective studies (3–6), the imaging diagnostic criteria for papillary lesions have not been unified. Diffusion-weighted imaging (DWI) is emerging as a favorable alternative for deriving perfusion information to complement dynamic contrast-enhanced magnetic resonance imaging of the breast. By calculating the apparent diffusion coefficient (ADC), DWI, which is sensitive to water diffusion, can provide a quantitative analysis of both the cellularity and perfusion of tumors and has the potential to provide an evaluation of lesion characterization. Hyunseok Seo reports that a high-resolution ADC map and a DWI can be accurately obtained by using isotropic diffusion-weighted imaging while reducing the artifacts caused by the diffusion anisotropy, compared to diffusion-weighted echo-planar-imaging (7). More other studies have already proved DWI and ADC values as promising tools in breast lesion detection, prognostic assessment, and therapeutic response prediction (8–10).

However, fewer studies were capable of proving DWI’s positive association with a diagnosis of breast papillary lesions, which contributed to the limited use of breast DWI in clinical practice. This retrospective study analyzes the mean ADC values observed from 94 different papillary breast lesions and aims to evaluate the role of ADC values in distinguishing malignant from benign lesions, especially in differentiating the histological subtypes of malignant lesions as well as in assessing the potential diagnostic contribution to papillary lesions in different enhancements.

## Materials and Method

### Data Collection

Clinical data were collected retrospectively on 69 female patients with papillary lesions who were admitted to our hospital from January 2021 to February 2022, with a total of 94 lesions. Among them, 51 cases were benign breast papillary lesions, all of which were IDP; 16 cases were borderline lesions, all of which were intraductal papilloma with ADH; and 27 cases were malignant lesions, including 13 cases of intraductal papilloma with DCIS, 3 cases of papillary DCIS, 1 case of EPC, 9 cases of SPC and 1 case of IPC. The inclusion criteria for this study were as follows: breast papillary lesions confirmed by postoperative pathology (one patient may have multiple lesions) and preoperative MRI examination was available from which the ADC values of the lesions corresponding to the postoperative pathology could be obtained on DWI. The exclusion criteria were as follows: lesions with non-high signal on DWI—namely, ADC values could not be obtained—and lesions with the coexistence of multiple pathological types, of which it was impossible to determine what kind of pathological type the ADC value belongs to.

### MRI Examination

Imaging was performed on the same 3T MR unit (Philips Ingenia). All patients were in the prone position. The Philips MRI scanning sequence included the following: (1) cross-sectional T2WI, using two-dimensional fast spin-echo sequence, SPAIR fat suppression, and the following scanning parameters: TR/TE, 5,000/65 ms; slice thickness/slice interval, 4/1 mm; FOV, 37.2 cm; matrix, 465 × 381; (2) cross-sectional diffusion-weighted imaging DWI, using single-shot SE-EPI sequence, NEX = 1, SPIR + SSGR fat suppression, *b* = 0, 800 s/mm^2^, and the following scanning parameters: TR/TE, 5,100/72 ms; layer thickness/layer spacing, 4/1 mm; FOV, 35 cm; matrix, 136 × 140; and (3) cross-sectional dynamic enhancement, three-dimensional gradient-echo sequence, and SPIR fat suppression. First, the plain scanned images were acquired and then collected by 4 to 5 consecutive phases without intervals after injecting the contrast agent (gadopentetate meglumine), followed by injection in the amount of 0.1 mmol/kg with a high-pressure syringe through the dorsal vein of the hand at a flow rate of 2.0 ml/s and then 15 ml of normal saline at the same flow rate. The scanning parameters were as follows: TR/TE, 4.2/2.1 ms; layer thickness/layer spacing, 1/0 mm; flip angle, 12°; FOV, 34 cm; and matrix, 407 × 404. Each scan lasted for 65 s. Imaging of all lesions was analyzed in consensus by two experienced breast radiologists. The solid area was selected at the layer with the largest diameter of the lesion to delineate the region of interest (ROI) on DWI corresponding to T2WI, dynamic enhancement, and subtraction images. The necrotic, cystic hemorrhagic parts of the lesion and where ROI was smaller than the range of the high signal area should be avoided as much as possible. The ADC value of the solid component of the lesion was measured on ADC maps.

### Statistical Analysis

Statistical analysis was performed using IBM SPSS 26.0 (the mean ADC value was made for lesions whose ADC values were presented as a range). The statistical diagram was performed by GraphPad Prism 8.4. *T*-test or one-way analysis of variance was used to compare the quantitative variables between two groups and the Bonferroni method for multiple comparisons. The receiver operating characteristic (ROC) curves were constructed to obtain the area under the curve (AUC) and the optimal threshold of the ADC value with its sensitivity and specificity for potential diagnosis contribution to papillary lesions. *P*-value <0.05 was considered statistically significant.

## Results

### Clinical Features

This study included a total of 94 papillary lesions of 69 patients ranging from 31 to 73 years old. The lesions were categorized as mass and non-mass enhancement according to the BI-RADS fifth edition (11). Among them, 54 cases were mass lesions, while 40 cases were non-mass lesions; 35 cases were lesions with diameters <1 cm, while the others were with diameters ≥1 cm. The general features of benign, borderline, and malignant lesions are summarized in **Table 1**.

**Table 1:** General features of benign, borderline, and malignant papillary breast lesions.

Groups	Benign	Borderline	Malignant	Total
Mean age (years old)	49.8	50.4	58.0	51.7
Number (cases)	51	16	27	94
Mass enhancement (cases)	35	7	12	54
Non-mass enhancement (cases)	16	9	15	40
Diameter, <1 cm (cases)	26	5	4	35
Diameter, ≥1 cm (cases)	25	11	23	59

### Comparison of Mean ADC Values in Benign, Borderline, and Malignant Papillary Lesions

**Figure 1:**
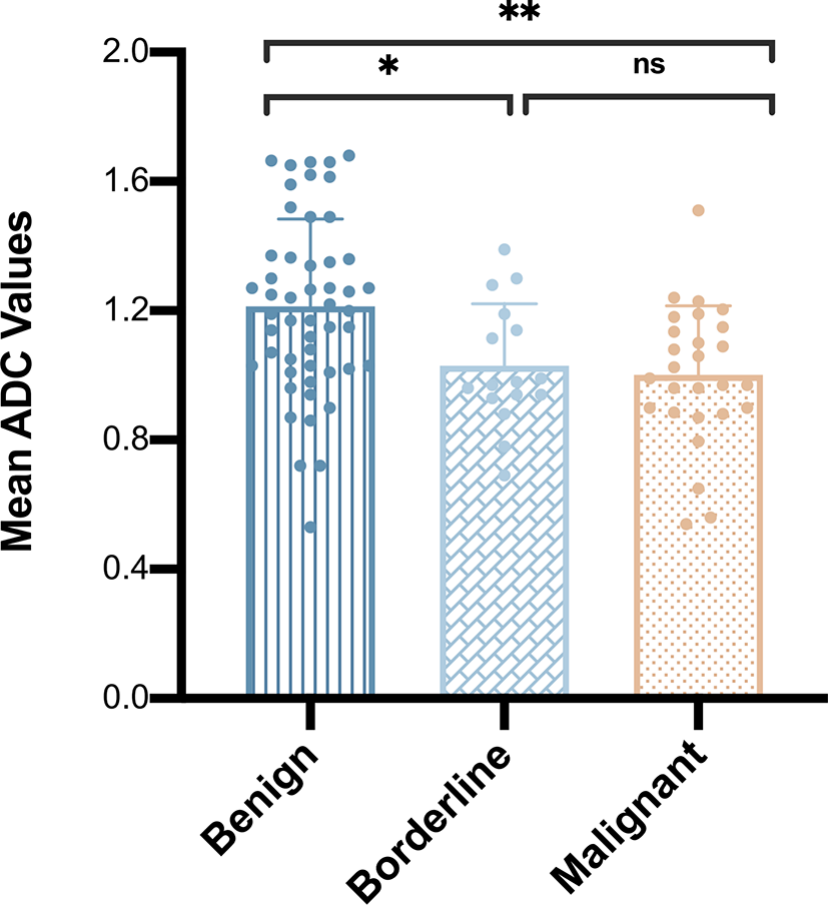
Comparison of mean apparent diffusion coefficient values among benign, borderline, and malignant papillary breast lesions. *P < 0.05; **P < 0.01; ns, *P* > 0.05.

The mean ADC values of benign, borderline, and malignant papillary lesions are shown in **Table 2**. The ADC values of benign papillary lesions (1.21 ± 0.27 × 10^-3^ mm^2^/s) were significantly higher than those of borderline and malignant papillary lesions (1.03 ± 0.19 × 10^-3^ mm^2^/s and 1.00 ± 0.21 × 10^-3^ mm^2^/s) (*P* < 0.05), while the ADC values proved no significant difference between borderline lesions and malignant lesions (*P* > 0.05) (**Figure 1**).

**Table 2:** Comparison of the mean apparent diffusion coefficient (ADC) values among benign, borderline, and malignant papillary breast lesions.

Papillary lesions	Number (cases)	Mean ADC values (×10^-3^ mm^2^/s)	*P*
Benign	51	1.21 ± 0.27	0.030^a^
Borderline	16	1.03 ± 0.19	1.000^b^
Malignant	27	1.00 ± 0.21	0.001^c^

a Compared to borderline lesions.

b Compared to malignant lesions.

c Compared to benign lesions.

**Figure 2:**
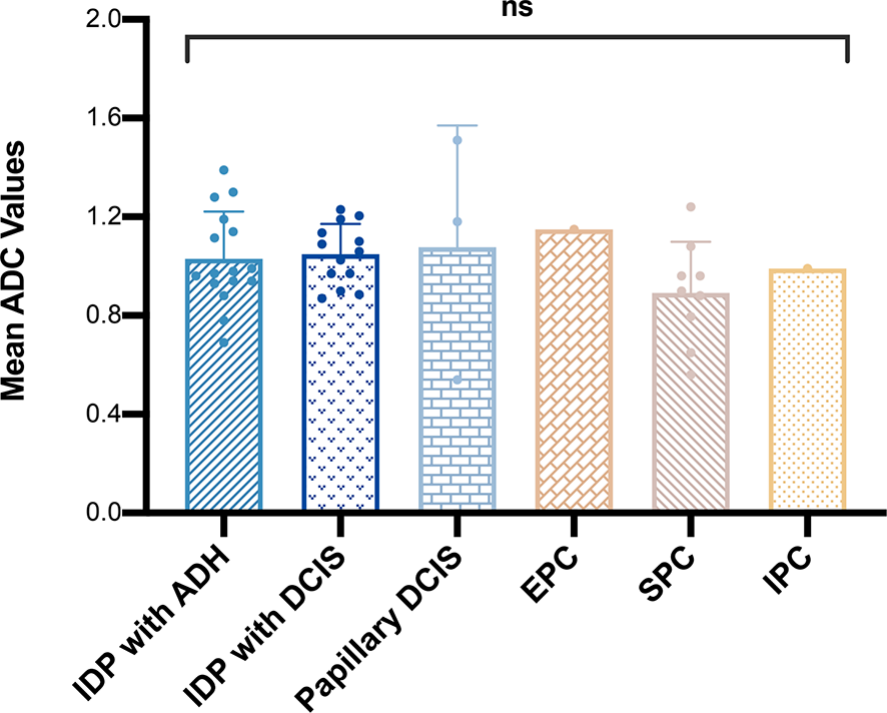
Comparison of mean apparent diffusion coefficient values among different malignant papillary breast lesion subtypes. ns, *P* > 0.05.

**Figure 3:**
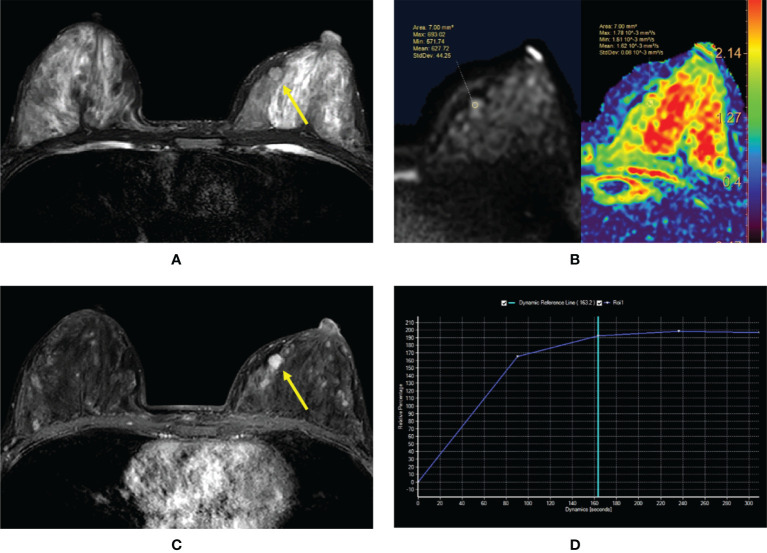
**(A–D)** Intraductal papilloma in a 38-year-old woman. **(A)** T2-weighted image showing an isointensity signal mass lesion (yellow arrow) in the left breast. **(B)** Diffusion-weighted imaging showing a hyperintensity signal and apparent diffusion coefficient (ADC) map showing mean ADC = 1.62 × 10^-3^ mm^2^/s. **(C)** Enhanced T1-weighted image showing a strong nodular enhancement (yellow arrow) with clear margins. **(D)** Time–signal intensity curve manifests as a rapid increase (initial phases) and a plateau type (delayed phases).

**Figure 4:**
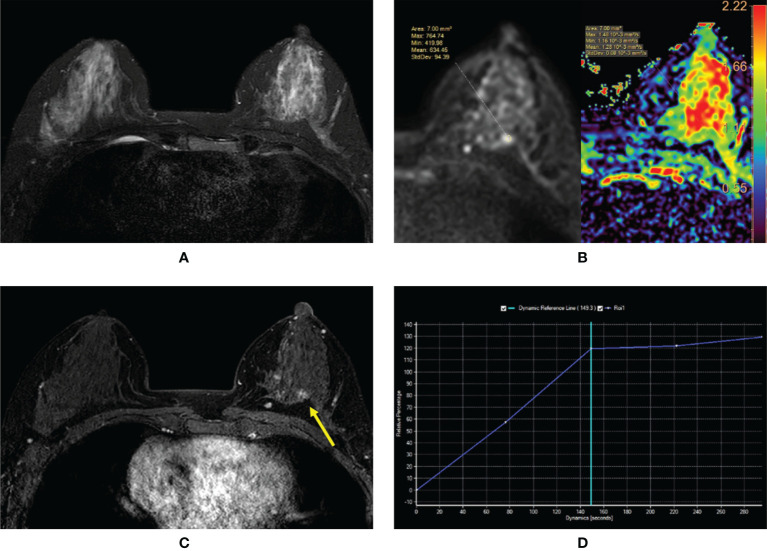
**(A–D)** Intraductal papilloma with atypical ductal hyperplasia in a 43-year-old woman. **(A)** T2-weighted image showing an isointensity signal and unclear lesion in the left breast. **(B)** Diffusion-weighted imaging showing a hyperintensity signal and apparent diffusion coefficient (ADC) map showing mean ADC = 1.28 × 10^-3^ mm^2^/s. **(C)** Enhanced T1-weighted image showing the nonhomogeneous enhancement of an irregular-shaped lesion with ill-defined margins (yellow arrow). **(D)** Time–signal intensity curve manifests as a rapid increase (initial phases) and a plateau type (delayed phases).

**Figure 5:**
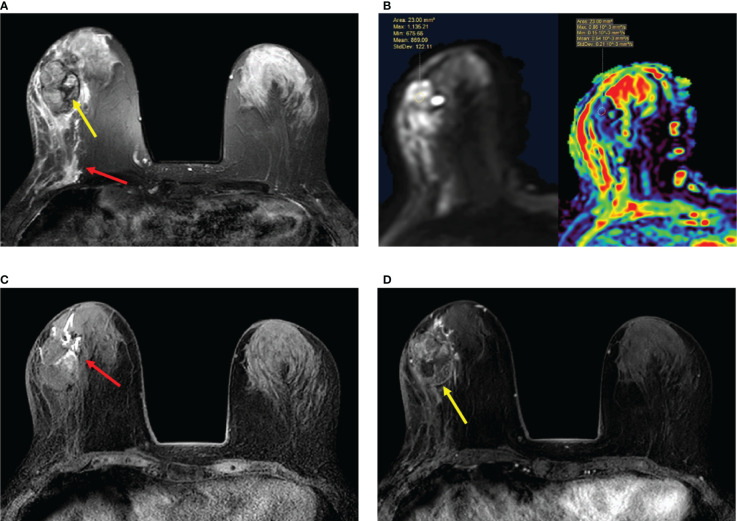
**(A–D)** Papillary ductal carcinoma *in situ* in a 72-year-old woman. **(A)** T2-weighted image showing a hypointensity signal mass lesion (yellow arrow) and a large edema signal behind the mass (red arrow) in the left breast. **(B)** Diffusion-weighted imaging showing a hyperintensity signal mass lesion and apparent diffusion coefficient map showing mean ADC = 0.54 × 10^-3^ mm^2^/s. **(C)** Plain T1-weighted image showing duct dilatation (red arrow) in front of the mass. **(D)** Enhanced T1-weighted image showing the nonhomogeneous enhancement of an irregular-shaped mass with ill-defined margins (yellow arrow). Time–signal intensity curve manifests as a slow increase (initial phases) and a persistent type (delayed phases).

In total, 13 cases of borderline papillary lesions were all intraductal papilloma with ADH, of which the mean ADC value was 1.03 ± 0.19 × 10^-3^ mm^2^/s. Among malignant papillary lesions, the mean ADC value of 13 cases of intraductal papilloma with DCIS was 1.05 ± 0.12 × 10^-3^ mm^2^/s, the mean ADC value of 3 cases of papillary DCIS was 1.08 ± 0.49 × 10^-3^ mm^2^/s, there was only 1 case of EPC and IPC each, and the ADC values were 1.15 × 10^-3^ mm^2^/s and 0.99 × 10^-3^ mm^2^/s respectively. SPC had the lowest mean ADC value which was 0.89 ± 0.21 × 10^-3^ mm^2^/s. However, there was no significant difference in the mean ADC values of borderline or any other malignant lesion subtypes (*P* > 0.05) (**Figure 2**). The MRI features of 3 different lesion subtypes are shown in **Figures 3**–**5**.

### ROC Curves for Papillary Breast Lesions

Therefore, our study categorized borderline lesions and malignant lesions as one group. The mean ADC value in borderline and malignant lesions was significantly lower than that in benign lesions (1.21 ± 0.27 × 10^-3^ *vs*. 1.01 ± 0.20 × 10^-3^ mm^2^/s, *P* < 0.05), and the differences between the mean ADC values of the two categories were statistically significant whether in mass or non-mass enhancement (*P* < 0.05) (**Table 3**).

**Table 3:** Comparison of mean apparent diffusion coefficient (ADC) values in different papillary breast lesion groups.

Groups	Mean ADC value (×10^-3^ mm^2^/s)		P
	Benign	Borderline and malignant	
All lesions	1.21 ± 0.27 (*n* = 51)	1.01 ± 0.20 (*n* = 43)	0
Mass enhancement lesions	1.16 ± 0.28 (*n* = 35)	0.97 ± 0.20 (*n* = 19)	0.011
Non-mass enhancement lesions	1.34 ± 0.21 (*n* = 16)	1.05 ± 0.21 (*n* = 24)	0

**Figure 6:**
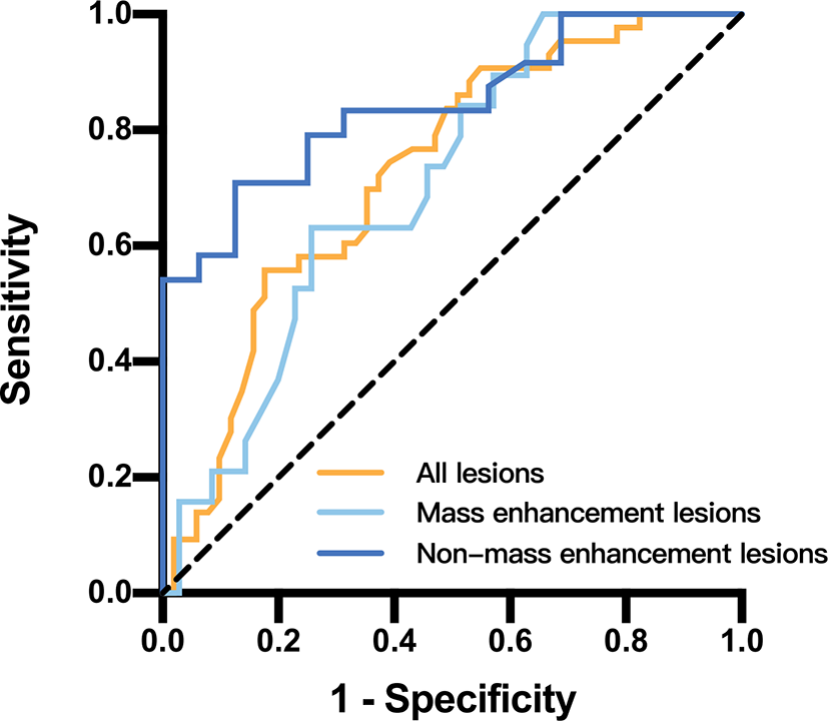
Receiver operating characteristic curves and area under the curve for papillary breast lesions in different papillary breast lesion groups.

The ROC curves and AUC for papillary breast lesions with different subtypes are presented on **Figure 6**. The threshold of ADC value to differentiate benign papillary breast lesions from malignant was 1.00 × 10^-3^ mm^2^/s (AUC, 0.728; sensitivity, 55.8%; specificity, 82.4%; *P* < 0.05). The threshold of the ADC value for mass lesions was 1.00 × 10^-3^ mm^2^/s (AUC, 0.706; sensitivity, 63.2%; specificity, 74.3%; *P* < 0.05), while for the non-mass lesions this was 1.14 × 10^-3^ mm^2^/s (AUC, 0.842; sensitivity, 70.8%; specificity, 87.5%; *P* < 0.05).

## Discussion

Papillary breast lesions had drawn increasing attention in clinical practice recently. Benign intraductal papillomas are currently recognized as premalignant lesions. The World Health Organization (WHO) classification of papillary breast lesions suggests that the risk of subsequent invasive breast cancer development in central papillomas without epithelial atypia is believed to increase to two times that of the general population while to three times that of peripheral papillomas (1, 12). It is strongly recommended to closely follow up through imaging examination for such benign lesions in the long term.

DWI is an advanced MRI technique that can measure the mobility of water molecules diffusing in tissue, which is impacted by biophysical characteristics such as cell density, membrane integrity, and microstructure of the breast. DWI is now widely used as an important addition to standard breast MRI protocol to screen early breast cancer and potentially predict the response to and monitor the effect of neoadjuvant treatment over time (8, 13). The ADC derived from DWI that provides a quantitative measure of observed diffusion restriction can be used to distinguish between benign and malignant breast lesions. Numerous studies have demonstrated significantly lower ADC values in malignant *versus* benign lesions (14). The ADC values of benign and malignant papillary breast lesions in this research were consistent with previous studies. The mean ADC value of benign papillary lesions (1.21 ± 0.27 × 10^-3^ mm^2^/s) was significantly higher than borderline lesions (1.03 ± 0.19 × 10^-3^ mm^2^/s) and malignant lesions (1.00 ± 0.21 × 10^-3^ mm^2^/s) (*P* < 0.05, respectively). We suggest that ADC values can also be used to differentiate between benign and malignant papillary lesions.

In our study, we achieved the optimal threshold of ADC value as 1.00 × 10^-3^ mm^2^/s through the ROC curve. The ADC value was the same as that what a meta-analysis based on 13,847 breast lesions concluded (15). Furthermore, this result from the meta-analysis was independent of Tesla strength, measure methods, and the choice of *b* values. In the study of Yildiz S et al. (16), the mean ADC values of benign and malignant papillary lesions were 1.339 × 10^-3^ and 0.744 × 10^-3^ mm^2^/s, respectively, with a threshold of around 0.859 × 10^-3^ mm^2^/s. The reason for the differences in results between the abovementioned research and our study lay in the fact that Yildiz S enrolled fewer papillary lesions (only 29 lesions), among which benign lesions took a big proportion (80%). Compared to his study, the ratio of benign and malignant lesions exhibited more reasonably in our research. We suggest that the optimal threshold of ADC value should be 1.00 × 10^-3^ mm^2^/s for discrimination of benign and malignant papillary lesions.

Papillary lesions of the breast represent diverse histological subtypes. Malignant lesion subtypes were difficult to distinguish through ADC values in our study (*P* > 0.05). Maric J et al. (17) also reported that there were no significant correlations between malignant lesion subtypes and ADC values. The highest ADC value of malignant pathology in our study attributed to EPC was 1.15 × 10^-3^ mm^2^/s, which did not correspond to the study of Tang WJ et al. (18). The mean ADC value in his study was 0.876 × 10^-3^ mm^2^/s based on 11 EPC lesions. SPC exhibited the lowest malignant pathology ADC values, which varied from 0.56 to 1.24 × 10^-3^ mm^2^/s, and the mean ADC value was 0.89 ± 0.21 × 10^-3^ mm^2^/s. The previous study (19) reported that the ADC values of SPC varied from 1.3 to 1.9 × 10^-3^ mm^2^/s. Several potential factors might explain the disparities between the results. Malignant papillary lesions represented heterogeneous histological subtypes that show various cellularity and vascularization causing different degrees of diffusion. ROI placement in two studies also significantly influenced the ADC values measured in breast tumors (20). We suggest that the performance of ADC to distinguish among these subtypes might be variable, and presumably more studies with larger cohorts from multiple institutions might be needed or it might be helpful to apply ADC dataset to machine learning techniques for lesion classification.

ADH occurring within an intraductal papilloma considered as a borderline lesion deserves increasing attention clinically of late for the risk of subsequent invasive breast cancer development in such lesion is believed to be increased to 7.5× that of the general population. The WHO Working Group’s classification of breast tumors defines atypical epithelial proliferation to be limited to <3 mm of extent as intraductal papilloma with ADH, whereas in intraductal papilloma with DCIS, it spanned ≥3 mm (21). There was no statistical significance of ADC value in differentiating between intraductal papilloma with ADH (1.03 ± 0.19 × 10^-3^ mm^2^/s) and with DCIS (1.05 ± 0.12 × 10^-3^ mm^2^/s) (*P* > 0.05) in our study. We presume that image examination such as MRI even with DWI is incapable of discriminating lesions of millimetric pathologic difference, especially between ADH and DCIS to date. We strongly recommend taking an active surgical procedure if any suspicious signs of ADH lesions are visible in MRI.

Correlations of ADC with discrimination of non-mass-like breast lesions had been inconsistent to date in conventional studies (22, 23). Wang LJ et al. (24) found that papilloma manifesting as non-mass enhancement (NME) could be due to the concomitant benign, atypical, and malignant proliferative lesions, and the ADC value showed no significant difference between benign and malignant NME papillary lesions. Our study demonstrated the diagnostic value of ADC to differentiate benign from malignant papillary lesions whether in mass enhancement or in non-mass enhancement. For the mass-enhanced lesions, the mean ADC values of benign and malignant lesions are 1.16 ± 0.28 × 10^-3^ and 0.97 ± 0.20 × 10^-3^ mm^2^/s, respectively, with a threshold of 1.00 × 10^-3^ mm^2^/s and diagnostic accuracy of 70.6%. For the non-mass-enhanced lesions, the mean ADC values of benign and malignant lesions are 1.34 ± 0.21 × 10^-3^ and 1.05 ± 0.21 × 10^-3^ mm^2^/s, respectively, with a threshold of 1.14 × 10^-3^ mm^2^/s and diagnostic accuracy of 84.2%. We confirm the positive association of ADC value with discrimination between benign and malignant lesions in both enhancements. The high performance of ADC will not be affected by the way lesions are enhanced.

In conclusion, the ADC value derived by DWI is capable of differentiating between malignant and benign papillary lesions. The optimal threshold of the ADC value can be 1.00 × 10^-3^ mm^2^/s. The ADC value is statistically significant in differentiating between benign and malignant papillary lesions whether in mass or non-mass enhancement. There is no statistical difference in the ADC value among histological subtypes of malignant lesions, and studies with larger patient groups are needed to assess the potential diagnostic performance. A surgical procedure should be performed at the first opportunity if any papillary lesion is diagnosed as a borderline lesion by MRI.

## Data Availability Statement

The raw data supporting the conclusions of this article will be made available by the authors without undue reservation.

## Ethics Statement

Written informed consent was obtained from the individual(s) for the publication of any potentially identifiable images or data included in this article.

## Author Contributions

WL, DZ, and PW designed the study. WL and DZ collected the data and performed the statistical analysis. WL and DZ reviewed the MR images. WG reviewed the pathology findings. DZ drafted the manuscript. WL revised the manuscript. All authors contributed to the article and approved the submitted version.

## Conflict of Interest

The authors declare that the research was conducted in the absence of any commercial or financial relationships that could be construed as a potential conflict of interest.

The reviewer L-MW declared a shared parent affiliation with the authors to the handling editor at the time of review.

## Publisher’s Note

All claims expressed in this article are solely those of the authors and do not necessarily represent those of their affiliated organizations, or those of the publisher, the editors and the reviewers. Any product that may be evaluated in this article, or claim that may be made by its manufacturer, is not guaranteed or endorsed by the publisher.
